# Cutaneous Functional Units Hierarchy, Version 2 Defined

**DOI:** 10.1093/jbcr/iraf156

**Published:** 2025-08-06

**Authors:** Ingrid S Parry, Miranda Yelvington, Janice F Bell, Reg Richard

**Affiliations:** Betty Irene Moore School of Nursing, University of California, Davis, 2570 48th Street Sacramento, CA 95817, United States; Department of Rehabilitation, Arkansas Children’s Hospital, 1 Children's Way Little Rock, AR 72202, United States; Betty Irene Moore School of Nursing, University of California, Davis, 2570 48th Street Sacramento, CA 95817, United States; Retired. Big Canoe, GA 30143, United States

**Keywords:** cutaneous functional units, skin, scar, burn, rehabilitation, cutaneokinematics

## Abstract

Cutaneous functional units (CFUs) offer considerable potential to enhance the burn community’s understanding of how burn injuries and resulting scars affect movement. However, the lack of a clear and comprehensive schema for calculating and locating CFUs has led to confusion and limited their applicability. The goal of this short communication is to provide an update on the CFU schema in table and figure format to encourage uniform use of a common model for consistent application of CFU principles and mapping in burn care and other professional fields concerned with skin movement.

## INTRODUCTION

Cutaneous functional units (CFUs)^1^ are familiar to the burn community, but the complexity of their defining schema limits their clinical utility and scientific progress on the topic.^2^^,^^3^ Cutaneous functional units represent the application of *Cutaneokinematics*—the biomechanical effects of dynamic skin movement—which holds great promise for advancing the burn community’s understanding of how burn injury and subsequent scars impact motion. The goal of this short communication is to propose a revised and clarified CFU hierarchical schema that can serve as a unified framework for integrating CFUs into future research and clinical practice. We also provide background contextual information and discuss key considerations and implications to support burn team members in maintaining fidelity to the original CFU principles.

## BACKGROUND

Richard et al. first identified and defined CFUs for 9 body areas where skin was recruited to accommodate motion at associated joint creases prone to developing burn scar contracture (BSC).^1^ Fundamentally, CFUs in their simplest form are used to quantify the number of potential BSC sites that a patient may develop. This “number count” is intended to assist burn clinicians in estimating the rehabilitation time needed for burn survivors’ recovery and in prioritizing treatment strategies aimed at preventing BSC and optimizing functional outcomes.

In 2015, 182 isolated and 401 nested CFUs were defined based on clinical observations and theory to form the CFU Hierarchy Version 1 (v1).^4^ This original version of the CFU schema was created for use in a specific multicenter study known as the ACT study.^5^ Subsequent studies utilized the CFU Hierarchy v1 to quantify the rehabilitation time and duration of orthotic use associated with prevention of BSCs.^6^^,^^7^ However, because only a summarized version of CFU Hierarchy v1 was published, confusion and miscalculations arose with attempts to map and quantify CFUs accurately.^3^ This lack of clarity highlights the importance of precise and consistent numeric representation of CFUs if they are to serve as a reliable quantitative measure in research.

Supporting exploration of the potential significance of CFUs in research, a recent study applied the CFU Hierarchy v1 for secondary analysis of the ACT dataset to investigate the relationship between burn characteristics within CFUs and motion limitations.^8^ This study found that the extent of burn and skin grafting within a CFU was significantly associated with reduced motion at hospital discharge. These findings validated the clinical relevance of CFUs and emphasized the importance of accurate and standardized CFU quantification when studying outcomes such as BSCs.

## CFU HIERARCHY

**Figure 1:**
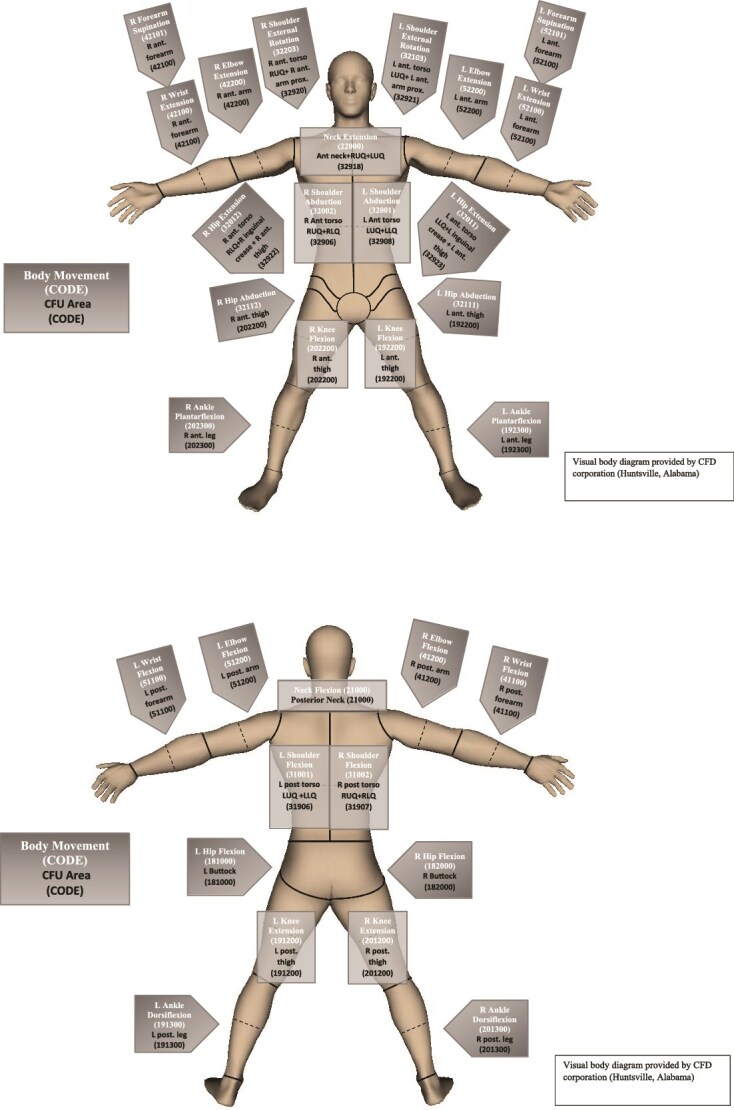

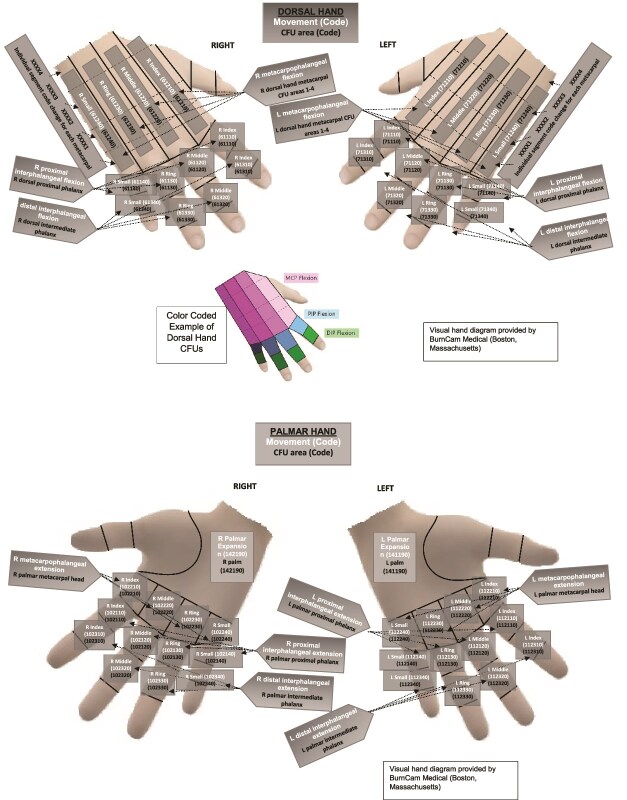

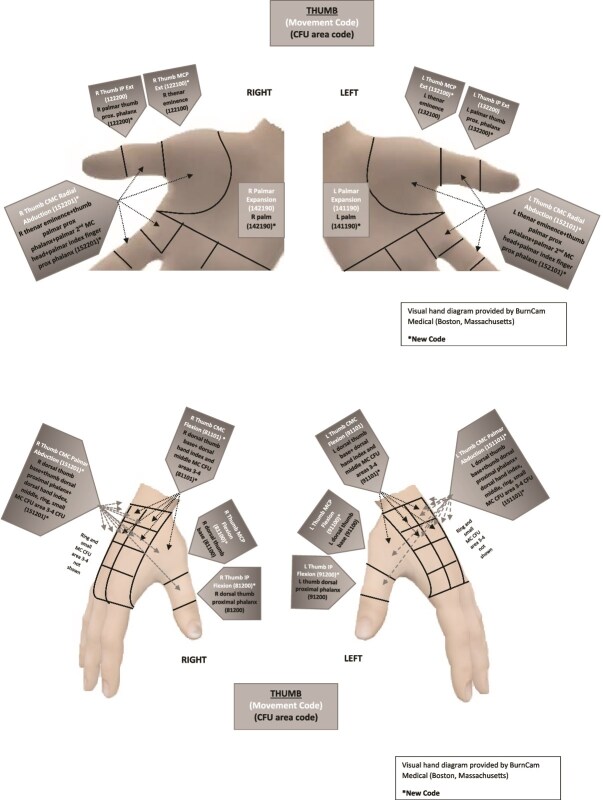
Cutaneous Functional Unit Codes and Linked Movements in Visual Diagram Format

During that recent study, several issues regarding the clarity and completeness of the CFU Hierarchy v1 were identified. Specifically, there was a lack of access to specific CFU codes associated with each motion, a need for combining certain original CFUs based on reevaluation of areas related to BSC, and the presence of joint motions without defining CFUs. In response, we have revised the CFU Hierarchy v1 and offer here a CFU Hierarchy Version 2 (v2) to clarify the coding schema and provide a detailed reference of the CFUs related to 100 motions. In this communication, the torso, extremities, and hands are included, but the head and associated facial movements are excluded because no additional research related to skin movement on the face has been conducted to make new recommendations. Codes and descriptors for primary and subdivided CFUs are provided in Table 1. As a visual representation, Figure 1 depicts the anatomical locations and groupings of the CFUs for each motion. Primary CFUs are defined as the largest defined area of skin closest to the skin crease where motion is being assessed. Defining CFUs using this hierarchical system enables CFUs to be subdivided or combined as needed. Therefore, the CFU areas that had other CFUs nested within the primary CFU in CFU Hierarchy v1 were coded and represented as Proximal CFU or Distal CFU relative to the joint crease (not anatomically proximal and distal) in the CFU Hierarchy v2. Proximal CFUs are defined as the portion of the primary CFU closest to the joint crease, and Distal CFUs are defined as the portion of the primary CFU farthest from the joint crease.^8^ Some primary CFUs did not have subdivided CFU codes, so proximal and distal CFUs could not be defined. Adjacent CFUs are defined as the smallest distinct skin unit immediately anatomically proximal to the primary CFU, and these can be visualized in Figure 1. Every motion also has a code, often the same code as the associated primary CFU. However, in cases where a given CFU is associated with multiple motions, a different motion code is named. The CFU code and motion code can now be easily linked.

**Table 1:** Joint Motions Associated with Primary, Proximal and Distal CFU Codes and Written Description of CFU Body Areas

	Side	Joint	Motion	Movement code	Primary CFU code	CFU area description (skin segment over the area described)	Proximal CFU code	Proximal CFU area description	Distal CFU code	Distal CFU area description
**Motions and CFUs originally defined in CFU Hierarchy v1**										
1	NA	Neck	Flexion	21 000	21 000	Posterior Neck (trapezius area)				
2	NA	Neck	Extension	22 000	32 918	Anterior neck + anterior torso RUQ + LUQ	22 000	Anterior neck	32 900	Anterior torso RUQ + LUQ
3	R	Shoulder	Flexion	31 002	31 907	R Posterior torso RUQ + RLQ	31 220	Posterior torso RUQ	31 210	Posterior torso RLQ
4	L	Shoulder	Flexion	31 001	31 906	L Posterior torso LUQ + LLQ	31 120	Posterior torso LUQ	31 110	Posterior torso LLQ
5	R	Shoulder	Abduction	32 002	32 906	R Anterior torso RUQ + RLQ	32 220	Anterior torso RUQ	32 210	Anterior torso RLQ
6	L	Shoulder	Abduction	32 001	32 908	L Anterior torso LUQ + LLQ	32 120	Anterior torso LUQ	32 110	Anterior torso LLQ
7	R	Shoulder	External Rotation	32 203	32 920	R Anterior torso RUQ + R anterior proximal arm	32 220	Anterior torso RUQ	42 220	R Anterior arm proximal
8	L	Shoulder	External Rotation	32 103	32 921	L Anterior torso LUQ + L anterior proximal arm	32 120	Anterior torso LUQ	52 220	L Anterior arm proximal
9	R	Elbow	Flexion	41 200	41 200	R Posterior arm	41 210	R Posterior arm distal	41 220	R Posterior arm proximal
10	L	Elbow	Flexion	51 200	51 200	L Posterior arm	51 210	L Posterior arm distal	51 220	L Posterior arm proximal
11	R	Elbow	Extension	42 200	42 200	R Anterior arm	42 210	R Anterior arm distal	42 220	R Anterior arm proximal
12	L	Elbow	Extension	52 200	52 200	L Anterior arm	52 210	L Anterior arm distal	52 220	L Anterior arm proximal
13	R	Forearm	Supination	42 101	42 100	R Anterior forearm	42 120	R Anterior forearm proximal	42 110	R Anterior forearm distal
14	L	Forearm	Supination	52 101	52 100	L Anterior forearm	52 120	L Anterior forearm proximal	52 110	L Anterior forearm distal
15	R	Wrist	Flexion	41 100	41 100	R Posterior forearm	41 110	R Posterior forearm distal	41 120	R Posterior forearm proximal
16	L	Wrist	Flexion	51 100	51 100	L Posterior forearm	51 110	L Posterior forearm distal	51 120	L Posterior forearm proximal
17	R	Wrist	Extension	42 100	42 100	R Anterior forearm	42 110	R Anterior forearm distal	42 120	R Anterior forearm proximal
18	L	Wrist	Extension	52 100	52 100	L Anterior forearm	52 110	L Anterior forearm distal	52 120	L Anterior forearm proximal
19	R	Hip	Flexion	182 000	182 000	R buttock				
20	L	Hip	Flexion	181 000	181 000	L buttock				
21	R	Hip	Extension	32 012	32 922	R anterior torso RLQ + R inguinal crease + R anterior proximal thigh	32 012	R inguinal crease	32 210	Anterior torso RLQ
22	L	Hip	Extension	32 011	32 923	L anterior torso LLQ + L inguinal crease + L anterior proximal thigh	32 011	L inguinal crease	32 110	Anterior torso LLQ
23	R	Hip	Abduction	32 112	202 200	R Anterior thigh	202 220	R Anterior thigh proximal	202 210	R Anterior thigh distal
24	L	Hip	Abduction	32 111	192 200	L Anterior thigh	192 220	L Anterior thigh proximal	192 210	L Anterior thigh distal
25	R	Knee	Flexion	202 200	202 200	R Anterior thigh	202 210	R Anterior thigh distal	202 220	R Anterior thigh proximal
26	L	Knee	Flexion	192 200	192 200	L Anterior thigh	192 210	L Anterior thigh distal	192 220	L and thigh proximal
27	R	Knee	Extension	201 200	201 200	R Posterior thigh	201 220	R Posterior thigh proximal	201 210	R Posterior thigh distal
28	L	Knee	Extension	191 200	191 200	L Posterior thigh	191 220	L Posterior thigh proximal	191 210	L Posterior thigh distal
29	R	Ankle	Dorsiflexion	201 300	201 300	R Posterior leg	201 310	R Posterior leg distal	201 320	R Posterior leg proximal
30	L	Ankle	Dorsiflexion	191 300	191 300	L Posterior leg	191 310	L Posterior leg distal	191 320	L Posterior leg proximal
31	R	Ankle	Plantarflexion	202 300	202 300	R Anterior leg	202 310	R Anterior leg distal	202 320	R Anterior leg proximal
32	L	Ankle	Plantarflexion	192 300	192 300	L Anterior leg	192 310	L Anterior leg distal	192 320	L Anterior leg proximal
33	R	Index MCP	Flexion	61 210	61 210	R Dorsal hand second metacarpal CFU area 1-4	61 211 + 61 212	R Dorsal hand 2nd metacarpal CFU area 1-2	61 213+ 61 214	R Dorsal hand second metacarpal CFU area 3-4
34	L	Index MCP	Flexion	71 210	71 210	L Dorsal hand second metacarpal CFU area 1-4	71 211 + 71 212	L Dorsal hand 2nd metacarpal CFU area 1-2	71 213+ 71 214	L Dorsal hand second metacarpal CFU area 3-4
35	R	Index MCP	Extension	102 210	102 210	R Palmar hand second metacarpal head				
36	L	Index MCP	Extension	112 210	112 210	L Palmar hand second metacarpal head				
37	R	Index PIP	Flexion	61 110	61 110	R Dorsal index finger proximal phalanx				
38	L	Index PIP	Flexion	71 110	71 110	L Dorsal index finger proximal phalanx				
39	R	Index PIP	Extension	102 110	102 110	R Palmar index finger proximal phalanx				
40	L	Index PIP	Extension	112 110	112 110	L Palmar index finger proximal phalanx				
41	R	Index DIP	Flexion	61 310	61 310	R Dorsal index finger intermediate phalanx				
42	L	Index DIP	Flexion	71 310	71 310	L Dorsal index finger intermediate phalanx				
43	R	Index DIP	Extension	102 310	102 310	R palmar index finger intermediate phalanx				
44	L	Index DIP	Extension	112 310	112 310	L Palmar index finger intermediate phalanx				
45	R	Middle MCP	Flexion	61 220	61 220	R Dorsal hand third metacarpal CFU area1-4	61 221 + 61 222	R Dorsal 3^rd^ metacarpal CFU area 1-2	61 223+ 61 224	R Dorsal third metacarpal CFU area 3-4
46	L	Middle MCP	Flexion	71 220	71 220	L Dorsal hand third metacarpal CFU area 1-4	71 221 + 71 222	L Dorsal 3^rd^ metacarpal CFU area 1-2	71 223+ 71 224	L Dorsal third metacarpal CFU area 3-4
47	R	Middle MCP	Extension	102 220	102 220	R Palmar hand third metacarpal head				
48	L	Middle MCP	Extension	112 220	112 220	L Palmar hand third metacarpal head				
49	R	Middle PIP	Flexion	61 120	61 120	R Dorsal middle finger proximal phalanx				
50	L	Middle PIP	Flexion	71 120	71 120	L Dorsal middle finger proximal phalanx				
51	R	Middle PIP	Extension	102 120	102 120	R Palmar middle finger proximal phalanx				
52	L	Middle PIP	Extension	112 120	112 120	L Palmar middle finger proximal phalanx				
53	R	Middle DIP	Flexion	61 320	61 320	R Dorsal middle finger intermediate phalanx				
54	L	Middle DIP	Flexion	71 320	71 320	L Dorsal middle finger intermediate phalanx				
55	R	Middle DIP	Extension	102 320	102 320	R Palmar middle finger intermediate phalanx				
56	L	Middle DIP	Extension	112 320	112 320	L Palmar middle finger intermediate phalanx				
57	R	Ring MCP	Flexion	61 230	61 230	R Dorsal hand fourth metacarpal CFU area 1-4	61 231 + 61 232	R Dorsal 4^th^ metacarpal CFU area 1-2	61 233 + 61 234	R Dorsal fourth metacarpal CFU area 3-4
58	L	Ring MCP	Flexion	71 230	71 230	L Dorsal hand fourth metacarpal CFU area 1-4	71 231 + 71 232	L Dorsal 4^th^ metacarpal CFU area 1-2	71 233 + 71 234	L Dorsal fourth metacarpal CFU area 3-4
59	R	Ring MCP	Extension	102 230	102 230	R Palmar hand fourth metacarpal head				
60	L	Ring MCP	Extension	112 230	112 230	L Palmar hand fourth metacarpal head				
61	R	Ring PIP	Flexion	61 130	61 130	R Dorsal ring finger proximal phalanx				
62	L	Ring PIP	Flexion	71 130	71 130	L Dorsal ring finger proximal phalanx				
63	R	Ring PIP	Extension	102 130	102 130	R Palmar ring finger proximal phalanx				
64	L	Ring PIP	Extension	112 130	112 130	L Palmar ring finger proximal phalanx				
65	R	Ring DIP	Flexion	61 330	61 330	R Dorsal ring finger intermediate phalanx				
66	L	Ring DIP	Flexion	71 330	71 330	L Dorsal ring finger intermediate phalanx				
67	R	Ring DIP	Extension	102 330	102 330	R Palmar ring finger intermediate phalanx				
68	L	Ring DIP	Extension	112 330	112 330	L Palmar ring finger intermediate phalanx				
69	R	Small MCP	Flexion	61 240	61 240	R Dorsal hand fifth metacarpal CFU area 1-4	61 241 + 61 242	R Dorsal 5^th^ metacarpal CFU area 1-2	61 243 + 61 244	R Dorsal fifth metacarpal CFU area 3-4
70	L	Small MCP	Flexion	71 240	71 240	L Dorsal hand fifth metacarpal CFU area 1-4	71 241 + 71 242	L Dorsal 5^th^ metacarpal CFU area 1-2	71 243 + 71 244	L Dorsal fifth metacarpal CFU area 3-4
71	R	Small MCP	Extension	102 240	102 240	R Palmar hand fifth metacarpal head				
72	L	Small MCP	Extension	112 240	112 240	L Palmar hand fifth metacarpal head				
73	R	Small PIP	Flexion	61 140	61 140	R Dorsal small finger proximal phalanx				
74	L	Small PIP	Flexion	71 140	71 140	L Dorsal small finger proximal phalanx				
75	R	Small PIP	Extension	102 140	102 140	R Palmar small finger proximal phalanx				
76	L	Small PIP	Extension	112 140	112 140	L Palmar small finger proximal phalanx				
77	R	Small DIP	Flexion	61 340	61 340	R Dorsal small finger intermediate phalanx				
78	L	Small DIP	Flexion	71 340	71 340	L Dorsal small finger intermediate phalanx				
79	R	Small DIP	Extension	102 340	102 340	R Palmar small finger intermediate phalanx				
80	L	Small DIP	Extension	112 340	112 340	L Palmar small finger intermediate phalanx				
**Motions originally coded to a CFU in CFU Hierarchy v1 but CFU area modified based on reevaluation and recoded for CFU Hierarchy v2**										
81	R	Thumb CMC	Flexion	81 100 *NEW CODE:* ***81 101***	81 100 *NEW CODE:* ***81 101***	R Dorsal thumb base *REDEFINED AREA: R dorsal thumb base + R dorsal hand index and middle metacarpal CFU area 3-4*				
82	L	Thumb CMC	Flexion	91 100 *NEW CODE:* ***91 101***	91 100 *NEW CODE:* ***91 101***	L dorsal thumb base *REDEFINED AREA: L dorsal thumb base + L dorsal hand index and middle metacarpal CFU area 3-4*				
83	R	Thumb CMC	Palmar Abduction	151 200 *NEW CODE:* ***151 201***	151 200 *NEW CODE:* ***151 201***	R dorsal first web space *REDEFINED AREA: R dorsal thumb base + R thumb dorsal proximal phalanx + R dorsal hand index, middle, ring, small metacarpal CFU area 3-4*				
84	L	Thumb CMC	Palmar Abduction	151 100 *NEW CODE:* ***151 101***	151 100 *NEW CODE:* ***151 101***	L dorsal first web space *REDEFINED AREA: L dorsal thumb base + L thumb dorsal proximal phalanx + L dorsal hand index, middle, ring, small metacarpal, CFUs area 3-4*				
85	R	Thumb CMC	Radial Abduction	122 100 *NEW CODE:* ***152 201***	122 100 *NEW CODE:* ***152 201***	R palmar thumb *REDEFINED AREA: R thenar eminence + R thumb palmar proximal phalanx + R palmar second metacarpal head + R palmar index finger proximal phalanx*				
86	L	Thumb CMC	Radial Abduction	132 100 *NEW CODE:* ***152 101***	132 100 *NEW CODE:* ***152 101***	R palmar thumb *REDEFINED AREA: L thenar eminence + L thumb palmar proximal phalanx + L palmar second metacarpal head + L palmar index finger proximal phalanx*				
87	R	Palmar	Expansion	142 190	142 100 NEW CODE: **142190**	*R ulnar palm area*				
88	L	Palmar	Expansion	141 190	141 100 NEW CODE: **141190**	*L ulnar palm area*				
**Motions originally coded to a CFU in CFU Hierarchy v1, but CFU numerically miscoded and therefore recoded for consistency with CFU Hierarchy v2**										
89	*R*	*Thumb IP*	*Extension*	*122 200*	*RECODED: **122200***	*RECODED: R thumb palmar proximal phalanx*				
90	*L*	*Thumb IP*	*Extension*	*132 200*	*RECODED: **132200***	*RECODED: L thumb palmar proximal phalanx*				
91	*R*	*Thumb MCP*	*Extension*	*RECODED: **122100***	122 100	R thenar eminence				
92	L	*Thumb MCP*	*Extension*	*RECODED: **132100***	132 100	L thenar eminence				
93	R	*Thumb MCP*	*Flexion*	*RECODED:* ***81 100***	*81 100*	*R dorsal thumb base*				
94	L	*Thumb MCP*	*Flexion*	*RECODED:* ***91 100***	*91 100*	*L dorsal thumb base*				
95	R	*Thumb IP*	*Flexion*	*RECODED:* ***81 200***	*81 200*	*R dorsal thumb proximal phalanx*				
96	L	*Thumb IP*	*Flexion*	*RECODED:* ***91 200***	*91 200*	*L dorsal thumb proximal phalanx*				
**Motions missing codes in CFU Hierarchy v1 and now included as new codes and defined areas in CFU Hierarchy v2**										
97	*R*	*Neck*	*Rotation*	*22 002*	*NEW CODE:* ***22 002***	*L half of 21 000 + L half of 22 000*				
98	*L*	*Neck*	*Rotation*	*22 001*	*NEW CODE:* ***22 001***	*R half of 21 000 + R half of 22 000*				
99	*R*	*Neck*	*Lateral flexion*	*22 012*	*NEW CODE:* ***22 002***	*L half of 21 000 + L half of 22 000*				
100	*L*	*Neck*	*Lateral Flexion*	*22 011*	*NEW CODE:* ***22 001***	*R half of 21 000 + R half of 22 000*				

Abbreviations: ant, anterior; CFU, cutaneous functional unit; DIP, distal interphalangeal; IP, interphalangeal; L, Left; LQ, lower quadrant; MC, metacarpal; MCP, metacarpophalangeal; post, posterior; PIP, proximal interphalangeal; R, Right; UQ, upper quadrant.

Shading indicates there is no subdivision of the CFU according to CFU Hierarchy v1.

## CONSIDERATIONS AND IMPLICATIONS FOR THE USE OF CFUS

Cutaneous functional units offer numerous promising avenues for future research, but the science of CFUs is only emerging. Both the original framework and the CFU Hierarchy v2 are defined through a combination of empirical testing of some skin areas^1^ and clinical observation and theory for other areas.^4^ We propose that for clarity and consistency, this CFU Hierarchy v2 be used as a common framework for future research, integrated into electronic burn diagrams, and iterated as evidence evolves.

Compared to TBSA, CFUs provide a more granular, location-specific, and functionally relevant prediction of motion outcomes.^9^ Because of their functional associations, CFUs offer a more meaningful metric that can potentially be used to determine needed therapy time and to guide resource utilization to best prevent BSCs.^6^^,^^7^ By identifying and quantifying injury characteristics, such as extent of burn and skin grafting within specific skin area recruited for functional movements, CFUs can inform both surgical and nonsurgical strategies to preserve or restore joint mobility, ultimately supporting more personalized and targeted care.

One roadblock to the full integration of CFUs into practice has been the lack of a consistent and supported CFU model. This CFU Hierarchy v2 now offers a model to allow consistent application of CFU principles and mapping. This is especially important for encouraging the continued evolution of CFU science and can facilitate transparent and replicable reporting of study methods, as well as improved comparison of findings across studies.

Another critical step toward enhancing the use of CFUs for both research and clinical care is their integration into electronic burn diagrams. Given the large number of CFUs, capturing and documenting the full burn injury involvement for each patient is time-consuming and labor-intensive. Automating the capture and quantification of burn characteristics within the CFU framework reduces the complexity and improves the feasibility of incorporating CFUs into routine clinical practice and research workflows. In addition, a structured coding system that integrates high-risk injury areas associated with impairment can support insurance coding and billing. To support this integration, we recommend that the CFU Hierarchy v2 be mapped directly onto existing and new electronic burn diagrams. To facilitate this adoption, we provide detailed descriptions of the codes and locations in Table 1 and Figure 1, which can be used by software developers to create standard models. This consistency will promote uniform data collection and interoperability across burn centers.

While the CFU Hierarchy v2 represents a step forward in the application of CFUs, it should not be viewed as a final endpoint, but rather as a common framework for continued investigation and a paradigm for clinicians and researchers to reference when seeking to understand and address the functional impact of scarring. Skin is a continuous structure and not segmented in the discrete ways the CFU Hierarchy v2 coding system might suggest. As such, more empirical research is still needed to better define the characteristics and locations of skin movement. By publishing the CFU Hierarchy v2, we provide an updated foundational framework that the scientific burn community can apply and refine as new evidence emerges.

## CONCLUSION

To date, CFU concepts have primarily been utilized and researched by burn therapists; however, cutaneokinematic principles extend beyond burn rehabilitation. This short communication is intended for all members of burn care teams, whether directly engaged in CFU research or studying related topics that may benefit from a shared framework, such as the CFU Hierarchy v2. In addition to its practical application within burn care, any discipline concerned with skin movement and its functional implications—including plastic surgery, dermatology, biomechanical engineering, and aeronautics—may find CFUs relevant. Advancing the understanding and application of CFUs will require a transdisciplinary approach that bridges clinical and scientific domains. To support this important trajectory of research, we present the CFU Hierarchy v2 as a tool that the scientific community can collectively utilize to advance its understanding of skin and scars and their impact on motion.

## Notes

Author contributions: Ingrid S. Parry (Conceptualization, Data curation, Formal analysis, Investigation, Methodology, Project administration, Resources, Software, Supervision, Validation, Visualization, Writing—original draft, Writing—review & editing [lead]), Miranda Yelvington (Conceptualization [equal], Methodology, Validation, Writing—original draft [supporting], Writing—review & editing [equal]), Janice F. Bell (Conceptualization, Data curation, Methodology, Project administration, Supervision [equal], Validation, Visualization [supporting], Writing—original draft, Writing—review & editing [equal]), and Reg Richard (Conceptualization, Data curation [equal], Methodology [supporting], Project administration, Resources, Supervision, Validation, Visualization, Writing—original draft, Writing—review & editing [equal])
